# The impact of end-of-life disability level on middle-aged and older adults’ utilization of medical services

**DOI:** 10.3389/fpubh.2025.1650570

**Published:** 2025-10-23

**Authors:** Xiaojing Huang, Wenqing Liu, Qi Pan, Man Yan, Lili Zhu, Chunxia Miao, Yun Zhao

**Affiliations:** 1 School of Management, Xuzhou Medical University, Xuzhou, Jiangsu, China; 2 Institution of Chinese Health Modernization, Xuzhou Medical University, Xuzhou, Jiangsu, China

**Keywords:** end of life, activities of daily living, medical services, middle-aged and older adults, Poisson regression, Tobit regression

## Abstract

**Background:**

As healthcare demands often intensify during the final stages of life, this study examines the intricate associations between disability severity and patterns of medical service utilization in end-of-life care.

**Methods:**

The data originate from the 2020 China Health and Retirement Longitudinal Study database (CHARLS). The activities of daily living scale and the instrumental activities of daily living scale are utilized to assess levels of functional disability. This cross-sectional study employed Poisson regression and Tobit regression to assess the influence of disability on the frequency and expense of outpatient and inpatient services utilized during the terminal phase.

**Results:**

The average scores of the activities of daily life and instrument activities of daily life were 10.83 ± 5.71 and 11.55 ± 5.88. The disability level of respondents in the end-stage of life was an important factor affecting the frequency of outpatient and inpatient medical services and inpatient service expenses, where the regression coefficients were 0.028 (*p* < 0.01), 0.014 (*p* < 0.05), and 1091.4 (*p* < 0.01), respectively. The partial marginal utility of the disability level for increasing inpatient expenses was 433.4 (*p* < 0.01).

**Conclusion:**

Severe disability levels predict an economic burden on families of respondents at the end of lives. This underscores the urgent necessity for targeted disability-focused interventions to alleviate economic burdens and ensure familial well-being.

## Introduction

During the final stages of life, middle-aged and older adults’ demand for medical services increases significantly as they cope with illnesses (1, 2). Middle-aged and older adults^1^ gradually encounter various disabilities as they age, making them increasingly reliant on the care of others (3–6). Some middle-aged and older adults may gradually lose their capacity to make critical decisions due to disability (7–9). The selection of medical care for terminally ill middle-aged and older adults often depends heavily on family members’ support and decision-making involvement (10, 11). However, families may encounter a dilemma when balancing the health needs of their middle-aged and older members against managing financial risks, and without careful consideration, they risk falling into a difficult and undesirable situation. As the global population ages, many families grapple with challenging end-of-life care decisions. Monitoring how families utilize health services during this critical period is crucial for safeguarding familial well-being.

As middle-aged and older adults near the end of their lives, does a disability result in excessive use of medical services, or does the disability hinder their ability to access adequate medical care? Existing studies have yielded insights into how disability influences access to and utilization of emergency and palliative care services. A U.S.-based study revealed that individuals with intellectual disabilities are more likely to receive end-of-life cardiopulmonary resuscitation (CPR), potentially because of lower rates of do-not-resuscitate (DNR) order utilization among this patient population (12). A German study highlighted that palliative care has disproportionately overlooked the needs of disabled individuals approaching the end of life (13). Consequently, there is a lack of consensus regarding whether the utilization of health services by middle-aged and older adults in the end stage of life, which is often characterized by disability, will increase or decrease.

Families and individuals often face information gaps when selecting medical services, with decision-making frequently shaped by economic constraints and cultural norms. For example, notable disparities were found in how residents of Eastern and Western countries engage in medical decision-making (14). In Eastern countries, including China, family members have a notably greater degree of involvement in medical decision-making processes (15). In reality, when confronted with such complex challenges, families seldom make optimal decisions. Research has shown that while financial incentive policies aimed at curbing excessive end-of-life medical care may reduce overutilization, they also risk inadvertently causing underutilization of necessary health care services (16). Nevertheless, analyzing the factors influencing families’ utilization of end-of-life medical services can inform evidence-based policies to mitigate health risks for both patients and their families.

Compared to developed countries, healthcare service utilization for terminal patients in China exhibits distinct comparative features. China’s end-of-life palliative care was initiated relatively late and encounters resource deficiencies of fundamental resources including funding, technology, and talent (17). In the high-income countries, continuity and coordination of care involve healthcare provisions from family to health facility throughout the life-course to provide a range of services (18). In terms of service model, China prioritizes disease treatment and life extension, while the development of comprehensive support services remains underdeveloped. Many other countries stress the integration of ‘holistic care’ and multidimensional service frameworks (19, 20). In terms of policy framework, China’s institutional development of end-of-life care is relatively slow and the standardization degree needs to be improved (21). Developed countries have established mature regulatory frameworks and quality assurance systems (22), underscoring the institutional divide between ‘developing and mature’.

China is experiencing rapid population aging, with 22% of its population projected to be over 60 years old by the end of 2024 (23). A study conducted in China indicated that an extension of lifespan may be accompanied by an increase in disability related to physical and cognitive functioning, particularly among frail older adults who survive with lingering health issues (24). Families typically hold primary decision-making authority over medical care for middle-aged and older members facing end-of-life health challenges (25). Studies have shown that the primary decision-maker in end-of-life care is typically the spouse or adult children (26–28). Families often prioritize exhaustive efforts to access and utilize medical services; this poses great economic risks for families and depletes health care resources for society. However, families may also risk underutilization of medical services due to economic constraints. Disagreements between numerous adult children and spouse in family decision-making often result in either the overutilization or underutilization of medical services. The use of medical services by Chinese families before the death of middle-aged and older adults is influenced by various factors.

Currently, global research on end-of-life medical care centres predominantly on addressing medical needs, emergency interventions, and palliative support. Few reports have examined the relationship between the utilization of medical services and factors influencing middle-aged and older adults at the end of their lives. Considering that disability may influence an individual’s medical decision-making, does disability lead to a decrease or increase in their utilization of medical services at the end of life? The evidence on how disability correlates with the utilization of end-of-life medical services can help identify which individuals require attention in their terminal stage, ensuring families’ basic welfare and mitigating economic risks they may face amid emotional distress. This study utilized a set of data from the China Health and Retirement Longitudinal Study (CHARLS) to describe the end-of-life disability status of older adults and its association with medical service utilization.

## Methods

### Study design and setting

CHARLS is a representative follow-up survey of people aged 45 and above in Chinese Mainland. For respondents who have passed away since their last visit in the fifth round of the survey (2020), a person familiar with their situation will complete the exit questionnaire. The sample from across the country gives the study a certain degree of robustness. This part of database focuses on capturing the utilization of health services and the disability status of adult individuals at the conclusion of their lives. Despite the COVID-19 pandemic during the 2020 data collection period, the mortality rate of China’s population exhibited no significant fluctuation. National Bureau of Statistics data indicate a 2020 death rate of 7.07‰ (9.98 million deaths), while the 2017–2019 period saw rates ranging from 7.06‰ to 7.09‰ (29). The national mortality rate in 2020 remained within this range and exhibited no significant fluctuations. We used the Strengthening the Reporting of Observational Studies in Epidemiology cross-sectional checklist when writing our report.

### Participants

This study employed the 2020 exit dataset from the CHARLS database, exclusively available in the 2020 Wave 5 survey. The exit database comprises a total of 770 individuals whose participation ceased due to death. Notably, eight participants were excluded from the analysis due to a lack of disability assessment data. In 2020, COVID-19 was expected to have a specific impact on the causes of death among residents. However, according to the CHARLS survey, only 2 out of 762 residents were hospitalized due to COVID-19. And only 16 residents reported access restrictions on seeking medical treatment as a result of epidemic prevention and control measures. Given this small proportion, we determined that their inclusion would have minimal impact on the results and thus did not exclude these two cases. Ultimately, data from 762 participants were utilized for analysis. The average age was 75.82 ± 10.80 years, and the male-to-female ratio was 1:0.73.

### Outcome variables

The measurement of medical service utilization encompassed four key indicators: the frequency of ambulatory medical service usage, the frequency of inpatient medical service usage, the expense of mobile medical services, and the expense of inpatient medical services. Outpatient services were assessed in the last month of life, while inpatient services were evaluated over the preceding year. Supplementary Table S1 presents descriptive statistics for the frequency and expense of medical services among participants during the end-of-life period.

### Independent variables

The Katz Index of Independence in Activities of Daily Living (ADL) and the Lawton Instrumental Activities of Daily Living (IADL) Scale have been used in studies as independent indicators for assessing BADL and IADL in Chinese older adults (30, 31). CHARLS 2020 measures the basic activities of daily living (BADLs) and instrumental activities of daily living (IADLs) of individuals in the 3 months prior to death. BADLs include 5 indicators: dressing, showering, eating, getting up, and using the toilet. IADLs also includes 5 indicators: cooking, shopping for groceries, making phone calls, taking medications, and managing money. Responses to each item were categorized into four levels: (1) No difficulty, (2) difficulty but can still perform the task, (3) difficulty and need assistance, and (4) unable to perform the task. The options are points numbered 1–4 in sequence order. The disability level is a comprehensive score calculated via these 10 indicators. Furthermore, it incorporates personal details such as demographic characteristics (sex, age, and chronic diseases), economic level (health insurance, current assets, real estate) and family support (number of offspring, whether having a spouse). The missing demographic information (gender and age) of certain the participants in the sample was supplemented by cross-referencing data from prior years. The number of offspring is operationalized as the number of biological children who were living at the time of data collection.

### Data analysis

In this study, a statistical description of the survey subjects’ disability levels in the 3 months preceding their death was conducted. Specifically, proportions were used to delineate the disability rates across 10 daily living ability indicators, whereas the mean ± standard deviation (SD) was used to characterize the average level of disability. GraphPad Prism 8.0 was used to generate a forest plot that illustrates the relationships between the 10 daily living ability indicators and the 4 health service utilization indicators. The values presented in the figure were calculated based on the coefficients obtained from univariate regression analysis, along with their corresponding 95% confidence intervals (CIs). Poisson regression was employed to investigate the relationship between service utilization frequency and its influencing factors. However, given the substantial proportion of zero values observed in ambulatory medical service utilization within this study, both zero-inflated Poisson regression and standard Poisson regression were utilized. Specifically, zero-inflated Poisson regression was applied to analyze the number of ambulatory medical services and inpatient services, with both models incorporating various independent variables. Notably, in the zero-inflated model, only constant terms were included, as no influencing factors were found to predict whether the number of ambulatory medical services utilized was zero. Furthermore, Tobit regression was utilized to analyze ambulatory medical expenses, inpatient expenses, and the associated independent variables. We implemented the interquartile range (IQR) method for robust outlier detection in the sample dataset, followed by systematic sensitivity analysis to validate the stability of our findings. Ultimately, the partial marginal utility (dy/dx) was computed to ascertain the average marginal impact of influencing factors on medical expenses in samples exhibiting actual expenses exceeding zero. All statistical tests were conducted via Stata 15.1. A *p* value less than 0.05 was considered significant.

## Results

### Daily living abilities in terminal life

Table 1 outlines the performance of ADLs among older adults during the last 3 months of life. Specifically, over 50% of the older adults retained fully functional independence in BADLs, which included dressing, eating, and using the toilet. Independent showering was achieved by only 41.99% of older adults, whereas 48.82% demonstrated the ability to arise from bed without assistance. Across the spectrum of functional independence—ranging from full autonomy to complete dependency—older adults who experienced difficulties yet managed to complete ADLs independently constituted the smallest subgroup, which was followed in increasing proportion by those requiring assistance, those unable to perform ADLs, and finally those reporting no difficulties at all. The overall activity rating for eating did not exceed 2 points. For the IADLs, over 57% of community-dwelling older adults maintained self-efficacy in making phone calls, taking medications, and managing money. Approximately 40.16% of older adults demonstrated independent proficiency in meal preparation, with a marginally higher proportion (43.83%) retaining functional autonomy in grocery shopping activities. The average of the IADLs score exceeded the average score of the ADLs (diff = −0.715 ± 0.297, *p* < 0.01).

**Table 1:** Functional level of BADLs and IADLs among community-dwelling older adults in the final 3 months of life.

BADLs and IADLs	The proportion of disability level				Mean ± SD
	1 (No difficulty)	2 (Difficult but still achievable)	3 (Have difficulties and need help)	4 (Unable to complete)	
Dressing up	53.41	6.17	16.54	23.88	2.11 ± 1.28
Showering	41.99	3.81	23.23	30.97	2.43 ± 1.31
Eating	59.71	5.91	15.22	19.16	1.94 ± 1.23
Getting up	48.82	7.61	20.47	23.10	2.18 ± 1.26
Using the toilet	50.00	5.91	20.87	23.23	2.17 ± 1.27
ADLs					10.83 ± 5.71
Cooking	40.16	2.49	7.35	50.00	2.67 ± 1.42
Shopping for groceries	43.83	1.57	8.40	46.19	2.57 ± 1.43
Making phone calls	58.53	1.44	7.74	32.28	2.14 ± 1.39
Taking medications	57.87	2.23	16.80	23.10	2.05 ± 1.29
Managing money	59.19	1.97	6.96	31.89	2.12 ± 1.39
IADLs					11.55 ± 5.88
Total score					22.38 ± 11.06

### Medical utilization in terminal care settings

**Figure 1:**
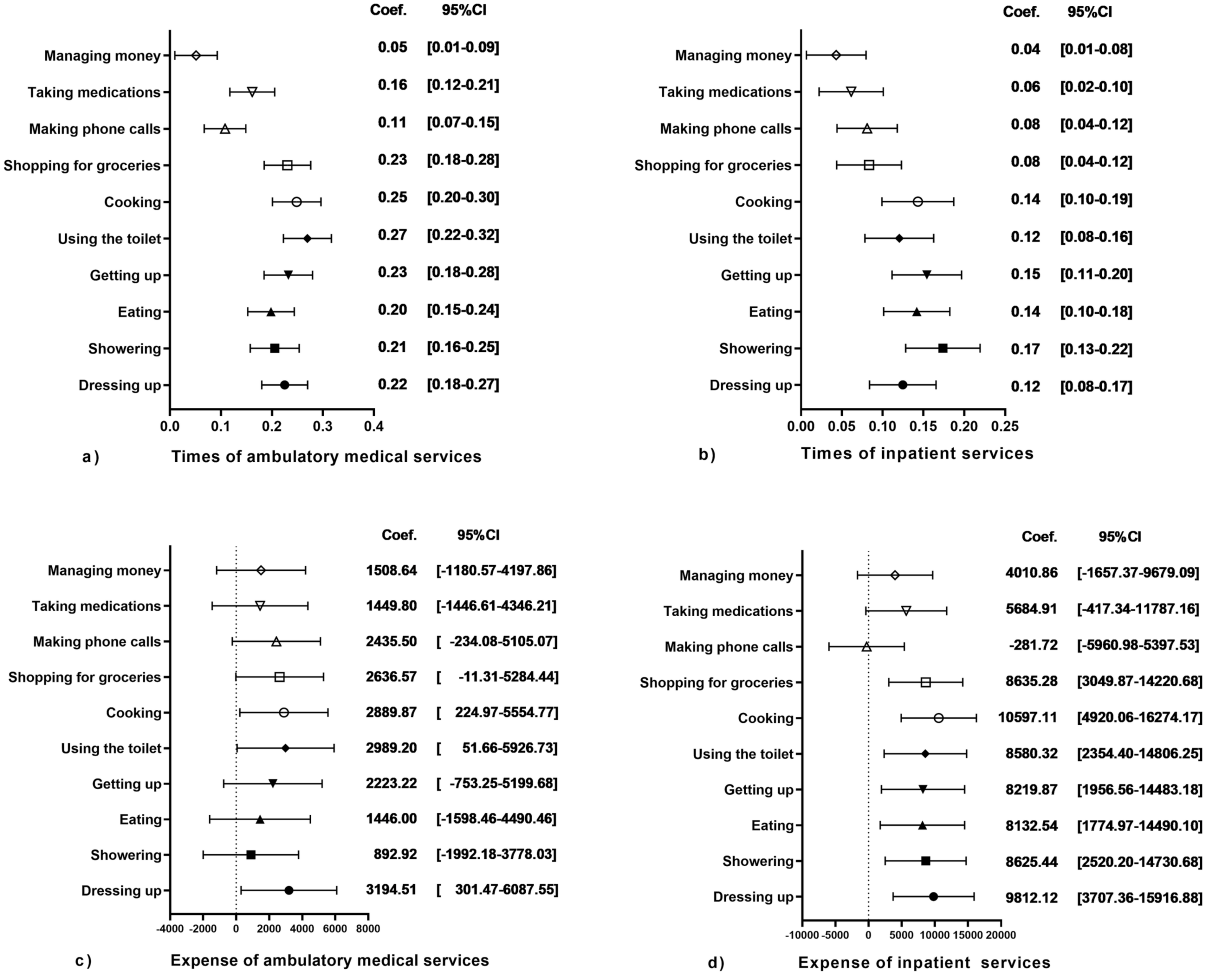
The forest plot illustrates the associations between functional capacity across 10 ADLs and medical utilization patterns among older adults. **(a)** Correlation Coefficients & 95% CIs: 10 ADLs vs. Times of Ambulatory Medical Services; **(b)** Correlation Coefficients & 95% CIs: 10 ADLs vs. Times of Inpatient services; **(c)** Correlation Coefficients & 95% CIs: 10 ADLs vs. Ambulatory Medical Expense; **(d)** Correlation Coefficients & 95% CIs: 10 ADLs vs. Inpatient Expense.

Figure 1 shows the associations between 10 daily living activities of older adults and medical utilization. Across all the assessed functional domains of daily living, retention of autonomy by middle-aged and older adults demonstrated a statistically significant positive association with ambulatory medical service utilization rates, with effect sizes shown in Figure 1a. As illustrated in Figure 1b, all assessed daily living activities demonstrated positive associations with inpatient service frequency in middle-aged and older adults. Ambulatory expenses were significantly associated exclusively with dressing up and cooking, and there were no discernible correlations with the remaining functional capacity indicators, as shown in Figure 1c. The inpatient service expense was significantly correlated with instrumental or basic activity capacity, except for making telephone calls, taking medication, and managing money, as shown in Figure 1d.

### Regression analysis of medical utilization

The results of the multivariate regression analysis of medical service utilization among older adults at the end of their lives are shown in Table 2. In Model 1, positive predictors included elevated disability level (*p* < 0.01), female sex (*p* < 0.05), owning properties (*p* < 0.01) and increased number of children (*p* < 0.01). Model 2 indicated that a high disability level (*p* < 0.01), health insurance coverage (*p* < 0.01), substantial current assets (*p* < 0.01), and having a spouse (*p* < 0.01) were associated with high utilization of inpatient services. In Model 2, the coefficient for current assets is statistically significant, but its value is extremely small, at just −0.00000245. Model 3 revealed that having chronic diseases (*p* < 0.05) and health insurance coverage (*p* < 0.05) were associated with high ambulatory medical expenses. Model 4 indicated that high inpatient expenses were significantly associated with elevated disability levels (*p* < 0.01), chronic diseases (*p* < 0.01), and substantial current assets (*p* < 0.05), whereas older age (*p* < 0.01) resulted in decreased inpatient expenses. Disability level is intimately linked to both ambulatory medical services and inpatient services utilization, as well as inpatient expenses, and serves as a pivotal factor influencing older adults’ utilization of medical services during the terminal stage of their lives. After sensitivity testing, the association between chronic disease status and ambulatory medical expense in Model 3 was no longer statistically significant (*p* < 0.05).

**Table 2:** Multivariate regression analysis of medical utilization among older adults in the terminal stage of life.

Variables	Model 1: Times of ambulatory medical services^‡^			Model 2: Times of inpatient services^‡^			Model 3: Ambulatory medical expense^‡^			Model 4: Inpatient expense^‡^		
	Coefficient	95%CI		Coefficient	95%CI		Coefficient	95%CI		Coefficient	95%CI	
Disability level	0.028**	**0.02**	**0.03**	0.024**	**0.01**	**0.02**	265.0	−75.6	605.7	1074.8**	**391.0**	**1758.6**
Sex												
Male	Reference											
Female	0.148*	**0.02**	**0.27**	−0.070	−0.17	0.07	3863.7	−3973.0	11700.4	5805.2	−10227.7	21838.1
Age	0.006	0.00	0.01	−0.008*	**−0.02**	**−0.01**	−179.6	−599.8	240.6	−1896.5**	**−2748.0**	**−1045.0**
Chronic diseases^†^												
No	Reference											
Yes	−0.070	−0.19	0.05	0.075	−0.09	0.12	8559.6*	**908.6**	**16210.6**	37042.8**	**21615.2**	**52470.5**
Health insurance												
No	Reference											
Yes	−0.312	−0.63	0.00	0.982**	**0.48**	**1.34**	30373.8*	**5187.0**	**55560.7**	32193.1	−7540.1	71926.3
Current assets	0.000	0.00	0.00	0.000**	**0.00**	**0.00**	0.0	−0.1	0.1	0.1*	**0.0**	**0.3**
Real estate												
No	Reference											
Yes	0.401**	**0.28**	**0.52**	−0.100	−0.26	−0.03	−7358.5	−15158.4	441.4	5757.3	−9884.4	21399.1
Number of children	0.098**	**0.05**	**0.14**	−0.003	−0.05	0.03	−192.5	−2895.0	2510.1	537.6	−4883.7	5958.9
Having a spouse												
No	Reference											
Yes	0.025	−0.12	0.17	0.238**	**0.13**	**0.39**	7681.4	−970.6	16333.3	16383.9	−848.7	33616.5
Vuong test	Z = 6.44 Pr > z = 0.00			Z = 5.39 Pr > z = 0.00			–	–		–	–	

† The category of chronic disease encompasses five conditions that have a profound impact on the health of Chinese residents: malignant tumours, chronic obstructive pulmonary disease, heart disease, stroke, and mental health problems.

‡ Model 1 and Model 2 employed zero-inflated Poisson regression, respectively. Models 3 and 4 utilized Tobit regression to accommodate censored cost observations.

* *p* < 0.05; ** *p* < 0.01. The bold values are statistically significant.

### Partial marginal utility

This study evaluated the partial marginal utility for variables with statistical significance. Table 3 indicates that the marginal contributions of chronic diseases and medical insurance to ambulatory medical service costs were 2005.6 (*p* < 0.05) and 4256.4 (*p* < 0.01), respectively. These contribution rates are very high. The marginal contributions of disability level, age, chronic diseases, and cash to inpatient expenses were 427.5 (*p* < 0.01), −754.3 (*p* < 0.01), 15460.4 (*p* < 0.01), and 0.1 (*p* < 0.05), respectively. The contribution rate of age was negative, which means that as age increases, the increase in inpatient expenses becomes negative, indicating a decrease in expenses.

**Table 3:** Partial marginal utility of important factors.

Expense	Factor	dy/dx	95%CI	
Ambulatory medical services	Chronic diseases	2005.6*	144.3	3866.9
	Health insurance	4256.4**	2317.2	6195.6
Inpatient services	Disability level	427.5**	156.0	699.0
	age	−754.3**	−1094.5	−414.2
	Chronic diseases	15460.4**	8721.0	22199.8
	Current assets	0.1*	0.0	0.1

* *p* < 0.05; ** *p* < 0.01.

## Discussion

Many middle-aged and older adults encounter varying degrees of disability preceding their death. The severity of a disability directly correlates with health outcomes, which can lead to increased demand for health care services. However, disability may also heighten the risk of exclusion from medical decision-making processes. This study seeks to investigate the use of healthcare services by Chinese families in individual end-of-life scenarios and its link to disability. This study revealed that end-of-life disability is intricately linked to health care utilization, with distinct patterns observed between outpatient and inpatient care services.

In ambulatory medical services, both 10 ADLs and composite disability levels among middle-aged and older adults demonstrate a dose–response relationship with health care utilization frequency. This association likely stems from China’s enhanced health care accessibility and optimized service delivery networks established through recent health system reforms. China’s National Health Commission reports that over 80% of residents have access to health care within 15 min (32). Families with patients can easily access ambulatory medical services. Among ADLs, only difficulties in dressing and cooking demonstrated a statistically significant association with higher outpatient expenses, whereas no such relationship was observed for other ADL domains. Consequently, the overall ADL level showed no statistically significant correlation with elevated outpatient expenses. This contrasts with data reported by other countries. A US study indicates that the overall cost of end-of-life outpatient care for cancer patients is rising (33). Given the study’s reliance on older family members’ recall of medical expenses, reported ambulatory medical expenses may exhibit recall bias, as lower payment amounts could increase the likelihood of underreporting.

Moreover‌, higher levels of disability are associated with increased utilization of end-of-life inpatient services and corresponding expenses. A previous study revealed that higher estimates that account for spending over the 12 months leading up to death more accurately reflect the full cost of a patient’s last year of life (34). This high expense likely arises from a misalignment between the services offered and the patient’s stated preferences (35). The progression of terminal illness in aging populations often necessitates a transition from ambulatory care to hospitalization due to acute care requirements beyond ambulatory service capabilities. The severity of disability is correlated with increased hospitalization frequency, potentially exacerbating health care expenditure burdens due to unmet communication needs. Health care policies that address financial risks associated with end-of-life care should prioritize forecasting inpatient expenditure patterns among middle-aged and older adults with severe disabilities to ensure equitable resource distribution. Previous research has confirmed that medical resource use by individuals tends to rise in the period preceding death (34, 36). A US study reveals that the principal increase lies in hospital service utilization (37). Studies from the US and Japan, respectively, indicate that serious illnesses and functional impairments drive the high cost of end-of-life care (38, 39). This study also corroborates this point. Regarding the high cost of end-of-life medical care, previous research indicates that either family intervention prior to an individual’s death or institutional end-of-life care can help reduce medical expenses and control costs (40). This service holds significant social value for China, a nation currently facing a deficit in palliative care services. The healthcare system must emphasize developing end-of-life care services tailored to Chinese families—with hospitals providing technical support, communities serving as operational hubs, and families forming the integral foundation for comprehensive end-of-life medical intervention services.

Demographic factors such as sex and age in older populations in the terminal life are distinctly associated with health care utilization patterns, with female patients exhibiting significantly higher ambulatory care rates than their male counterparts. This disparity may be attributable to gendered disparities in preferences for specific modalities of health care delivery. In China, females and older adults are more likely to choose local, lower-quality community health service institutions (41). The finding indicates that women exhibit a notable preference for the accessibility of medical services. This research reveals an inverse relationship between advancing age and hospitalization expenditure metrics. Previous studies established that middle-aged and older adults exhibit a heightened propensity to report discordant treatment decisions or opt for treatment de-escalation as they advance into more senior age categories (42, 43). Expense of inpatient service decreases with increasing age among older adults, which is consistent with documented age-related health care utilization patterns.

Terminal-stage middle-aged and older adults with chronic conditions demonstrate significantly higher outpatient and inpatient costs, coupled with substantial marginal cost contributions. Chronic disease is a significant determinant of recurrent medical service utilization in both emergency departments and inpatient care settings (44, 45). Obviously, this situation is more prominent at the end of life. The existing literature confirms heightened health care service utilization during the terminal year of life among patients with chronic diseases, with the majority of aggregate health care expenditures attributable to inpatient care services (46, 47).

The expense of outpatient services by medically insured middle-aged and older adults approaching the end of their life has increased, whereas inpatient service use has concomitantly increased. This discrepancy arises because, under China’s basic medical security system, outpatient payments are predominantly based on fee-for-service (FFS) models, whereas inpatient services are reimbursed using Diagnosis-Related Groups (DRGs). Consequently, in outpatient settings, insured residents may exhibit a higher propensity to utilize services due to reduced out-of-pocket costs associated with coverage (48). Concurrent with the expansion of China’s DRG-based hospitalization payment reforms, health care providers are incentivized to fragment continuous inpatient episodes into multiple admission events as a cost-containment strategy (49). Ambulatory care settings may absorb cost burdens previously borne by inpatient services, as evidenced by the migration of treatment and pharmaceutical revenue streams from inpatient to outpatient departments (50, 51). This provides a plausible mechanism for elevated end-of-life outpatient costs among insured middle-aged and older adults. Previous research has substantiated the substitution effect between outpatient and inpatient service utilization, demonstrating that patients in regions with higher out-of-pocket costs for outpatient care exhibit a reduced likelihood of seeking outpatient treatment and an increased tendency to opt for more expensive hospitalization services (52).

In this study, no statistically significant correlation was observed between spousal presence and medical service utilization. This may be attributed to the fact that the majority of research subjects are individuals aged 60 years and older. Confucian family norms, which are deeply embedded in Chinese cultural traditions, significantly influence end-of-life care decision-making processes and service utilization patterns among older populations, which contrasts sharply with the individualistic care paradigms that characterize Western health care systems (53). Therefore, in China, the choice of medical services for terminally ill parents largely depends on their children’s decisions. The research indicates that, regardless of whether one child or multiple children are involved, there is no difference in the utilization of end-of-life hospitalization medical services among older adults. However, the more children there are, the more often ambulatory medical services are utilized. This observation may stem from the availability of robust familial support networks, which enable the effective utilization of ambulatory care resources for managing less severe health conditions. In cases of severe health issues requiring hospitalization, the final decision-making authority rests with physicians rather than family members.

This study draws on cross-sectional data from a nationwide sample of end-of-life disability assessments in China, showcasing methodological strength with public health relevance. The research design investigates how difficulties in ADLs influences medical service utilization among end-of-life patients. Sampling exhibits representativeness, while statistical methods employ multivariate models to control for confounding factors, ensuring robustness in causal inference. Though cross-sectional designs inherently limit time-series causality verification, sensitivity analyzes strengthen result credibility.

### Limitations

The limitations of this study mainly stem from the acquisition of data and methodology. Because the sample is mostly composed of Chinese individuals, the results may not be generalizable to other racial and ethnic groups. The collected data come from the memory of family members of older adults, so there is inevitably a certain degree of recall bias. Although external environmental factors, such as the service support provided by local medical institutions, may still influence the utilization of end-of-life medical services, two key points must be highlighted. First, China’s medical industry adheres to a nationally unified model, operating as a public service sector led by the government. Second, due to data constraints, the model does not incorporate information on variations in the number of industries within the specific external environments of the research subjects. This study employed a cross-sectional research design, and the observed associations between variables must be interpreted with caution, as the methodological approach precludes the establishment of causal relationships. Finally, medical expenses remained unadjusted for inflation or regional differences, owing to the structure of the healthcare service system and the prevailing inflation level.

## Conclusion

Disability exerts differential influences on both ambulatory and inpatient medical service utilization among terminal middle-aged and older populations, with variations observed in utilization frequency and expenditure levels. This study demonstrated that Chinese households significantly increase medical service utilization in response to functional disabilities in dying family members. Health care policy development should prioritize end-of-life care for severely impaired middle-aged and older adults, as their conditions often correlate with heightened health care demands at the family level.

To proactively address population aging in China, we advocate institutionalizing the development of China’s palliative care service system. This includes establishing comprehensive home-based care services and expanding basic medical insurance coverage for palliative care to enhance quality of life and welfare for terminally ill individuals and their families. A critical avenue for future research lies in defining family medical intervention needs for terminally disabled individuals through disability assessment—an important dimension requiring continued exploration.

## Data Availability

Publicly available datasets were analyzed in this study. This data can be found here: http://charls.pku.edu.cn/en/.

## Footnotes

^1^The National Bureau of Statistics of China categorizes the age range of 45–59 as the middle-aged stage, and those aged 60 and above as older adults.
