# Supplementary material for: The impact of end-of-life disability level on middle-aged and older adults’ utilization of medical services
Source: Front Public Health. 2025 Oct 23;13:1650570. doi: 10.3389/fpubh.2025.1650570 (PMC12588994; doi:10.3389/fpubh.2025.1650570)
Supplement: Supplementary file 1 [file Image_1.pdf]

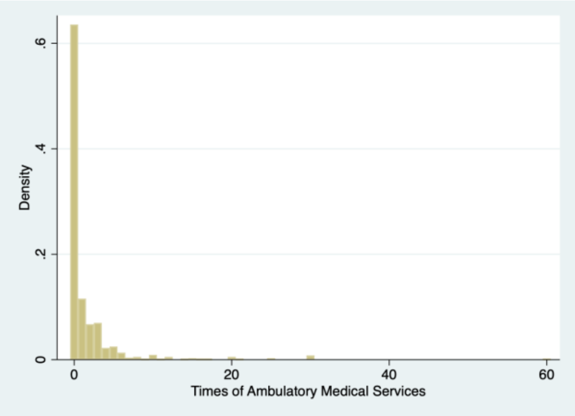

a) Frequency distribution of ambulatory medical services

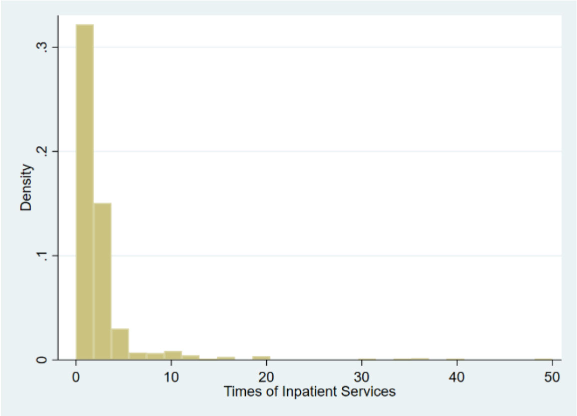

b) Frequency distribution of inpatient services

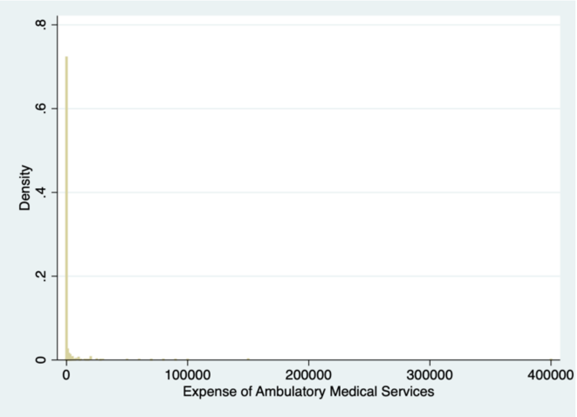

c) Frequency distribution of ambulatory medical expense

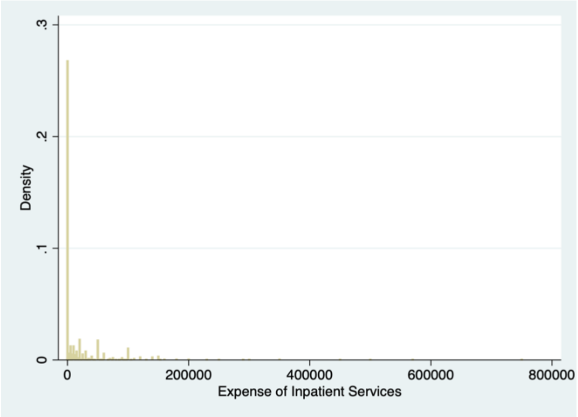

d) Frequency distribution of inpatient expense

Figure S1 the descriptive statistics for the times and expense of medical services among middle-aged and older adults during the end-of-life period
